# Supplementary material for: Moving towards culturally competent health systems for migrants? Applying systems thinking in a qualitative study in Malaysia and Thailand
Source: PLoS One. 2020 Apr 6;15(4):e0231154. doi: 10.1371/journal.pone.0231154 (PMC7135217; doi:10.1371/journal.pone.0231154)
Supplement: S1 File — (DOCX) [file pone.0231154.s001.docx]

**S1. Transcription guide**

| (word) | Word(s) in round brackets indicate transcriber guess at unclear word |
| --- | --- |
| CAPITALS | Words spoken more loudly; being emphasised |
| (…) | Indicates unclear material omitted by transcriber |
| [ ] | Square brackets enclose material added by author |
| … | Indicates material omitted by author |
| – | Change of thoughts or a pause |
| Ref: Green, J. & Thorogood, N. (2009). Qualitative Methods for Health Research. London: SAGE. | |
