# Supplementary material for: Moving towards culturally competent health systems for migrants? Applying systems thinking in a qualitative study in Malaysia and Thailand
Source: PLoS One. 2020 Apr 6;15(4):e0231154. doi: 10.1371/journal.pone.0231154 (PMC7135217; doi:10.1371/journal.pone.0231154)
Supplement: S2 File — (DOCX) [file pone.0231154.s002.docx]

**S2. Topic guides**

**MALAYSIA**

**A. Migrant workers**

Interview topics and questions that form the broad framework of discussion with migrant workers will include:

**Topics:**

- Knowledge and perception of healthcare services available
- Experience with access to healthcare in Malaysia
- Experience with barriers to access to healthcare in Malaysia
- Experiences with employers in relation to illness or injury
- Experience with healthcare workers with regards to healthcare treatment
- Suggestion for improvement in health policy or services available for migrants

**Introductory questions:**

1. Sex (M/F)
2. Date of Birth
3. Nationality
4. Years in Malaysia
5. Which country did you leave to come here?
6. What do you work as?
7. Are you employed by an individual or a company?

**Open questions:**

1. What are the most common health problems that you or your friends have faced during your stay in Malaysia? (examples)
2. Can you tell me where you or your friends will go for healthcare services when you are ill in Malaysia?
3. Can you share with me what is the healthcare experience like for migrant workers in Malaysia?

Prompts: How? Availability? Experience? Case studies?

1. What do you think regarding to healthcare services in Malaysia?

Prompts: awareness of services available for migrants, insurance schemes, and injury compensation schemes

1. What are the difficulties you or your friends have faced in accessing care?

What are the key barriers for migrant workers in accessing care in Malaysia?

Prompts: Barriers from individual, health system, community/cultural, stigma, geographical, financial, immigration status, fear of deportation? Case studies?

1. How do you pay for health care services?

What are the financial barriers for migrant workers in accessing healthcare in Malaysia?

Prompts: Are you covered by any insurance scheme? Does your employer pay your medical bills? Can you afford to seek treatment? Does seeking healthcare cause you financial hardship? Case studies?

1. What are your experiences with your employer/employers in relation to access to healthcare for illness or injury?

What is the experience of migrant workers with employers with regards to healthcare treatment?

Prompts: Are employers supportive? Will pay be docked for non-attendance? Will the worker be fired? Will employer pay for healthcare? Case studies?

1. What is your experience with healthcare workers with regards to healthcare treatment?

Prompts: positive/negative? Are they friendly? Communication barrier? Cultural appropriateness? Stigma? Case studies?

1. What are your suggestions for the improvement in health policy or services available for migrants in Malaysia?

**B. Key informant interviews: NGOs, migrant representatives, trade unions, academia**

Interview topics and questions that form the broad framework of discussion on policy protecting the health of migrants will include:

Topics:

- Knowledge of healthcare policy and services available in Malaysia
- Experience with migrant access to healthcare in Malaysia
- Perceptions or experience of barriers to migrant access to healthcare in Malaysia
- Experiences with employers of migrants in relation to work related illness or injury
- Experience of migrants with healthcare workers
- Suggestion for improvement in health policy or services available for migrants

**Introductory questions**

For representatives of migrant workers communities:

1. What is your role in your community/organisation?
2. What communities or nationalities does your organisation represent?
3. What is the demographic profile of migrants in your community?
   (age, sex, occupation, marital status)
4. What is immigration status of the communities that your organisation represents? (documented/undocumented, economic migrants, refugees, stateless people)

**Open questions**

1. Can you tell me about healthcare policy and services available for migrant workers in Malaysia?

Prompts: awareness of services available for migrants, insurance schemes, and injury compensation schemes

1. Could you please share experience of migrant access to healthcare in Malaysia?

What is the healthcare experience like for migrant workers in Malaysia?

Prompts: Where? How? Availability? Experience? Case studies?

1. Could you please share experience of barriers to access to healthcare of migrant workers in Malaysia?

What are the key barriers for migrant workers in accessing care in Malaysia?

Prompts: Barriers from individual, health system, community/cultural, stigma, geographical, financial, immigration status, fear of deportation? Case studies?

1. What are the perceived barriers to access to healthcare for migrant workers in Malaysia

What are the key barriers for migrant workers in accessing care in Malaysia?

Prompts: Barriers from individual, health system, community/cultural, stigma, geographical, financial, immigration status, fear of deportation? Case studies?

1. What kind of healthcare facilities do migrants go to when they are ill?

Prompts: Public or private? And why? Do many opt not to seek care?

1. How do migrants pay for healthcare?

What are the financial barriers for migrant workers in accessing healthcare in Malaysia?

Prompts: Do you know migrants covered by insurance schemes? Do employers pay for medical bills? Can migrants afford to seek treatment? Does seeking healthcare cause financial hardship? Case studies?

1. What are migrants experience with employers when they are ill?

What is the experience of migrant workers with employers with regards to healthcare treatment?

Prompts: Are employers supportive? Will pay be docked for non-attendance? Will the worker be fired? Will employer pay for healthcare? Case studies?

1. What are migrants experience with healthcare workers?

Prompts: positive/negative? Are they friendly? Communication barrier? Cultural appropriateness? Stigma? Case studies?

1. How the organization (you are representing) involved in migrant health issue? What is your organization’s role?

Prompts: What are the organization’s aims and activities relating migrant access to health care? What are the key success and challenges of your organization while working on migrant health issue? What is your opinion on collaboration among different sectors working on migrant health issue?

1. What are your suggestions to improve health policy and services for migrants in Malaysia?

**C. Medical doctors/Health workers**

Interview topics and questions that form the broad framework of discussion on policy protecting the health of migrants will include:

Topics:

- Knowledge of healthcare policy and services available in Malaysia
- Experience with migrant access to healthcare in Malaysia
- Perceptions or experience of barriers to migrant access to healthcare in Malaysia
- Experiences with employers of migrants in relation to work related illness or injury
- Experience of healthcare workers with migrants
- Suggestion for improvement in health policy or services available for migrants

**Introductory questions**

1. In what capacity do you deal with migrant workers?
2. What is immigration status of the communities that you see? (documented/undocumented, economic migrants, refugees, stateless people)
3. What are the demographic characteristics of the migrant workers you see? (male/female, occupation, country of origin)

**Open questions**

1. Do you have difficulty communicating with migrant workers? How do you overcome this?
2. What are the common conditions that migrant workers present with?
3. Who pays for migrant’s health services? Migrant/ employer
4. Do healthcare services for migrant workers cost more than Malaysians?
5. What is your opinion of migrant workers knowledge/awareness on healthcare issues?
6. Can you tell me about healthcare policy and services available for migrant workers in Malaysia?

Prompts: awareness of services available for migrants, insurance schemes, and injury compensation schemes

1. Could you tell me about FOMEMA services?
2. What is your opinion of occupational health and safety measures taken by migrant workers and their employers?
3. Do you provide sexual reproductive health services for migrants? E.g. contraception
4. Do you have any suggestions for future improvement for migrant workers in healthcare?

**THAILAND**

**For migrant health volunteers and migrant health workers (MHVs/MHWs):**

| **Topics** | **Questions** |
| --- | --- |
| **Motivation** | - Why did you decide to become a MHV/MHW? Or - - Why did you decide to quit being a MHV/MHW? |
| **Roles/Responsibilities** | - What is your opinion about MHV/MHW roles and responsibilities? |
| **Organizational support** | - What is your opinion about the process of MHVs/MHWs selection? - What is your opinion about supervision from organizations, including mentoring, training? - What is your opinion about renumeration of MHVs/MHWs? |
| **Opinions about strengths/challenges and suggestions** | - Besides language, what are the main cultural misunderstandings between migrant patients and health workers here?   Prompts: patient understanding about diseases, traditional medicine use, treatment adherence (differences by nationality?)   - What kinds of problems do you have while working as MHVs/MHWs? If specified, how do you solve these problems? - What is your opinion about MHV/MHW services? How can your work be better supported? |

**For health personnel and chief of organizations/Non-Government Organizations/Other supporting organizations for MHVs/MHWs services:**

| **Topics** | **Questions** |
| --- | --- |
| **Background and structure** | - Does your organization have migrant-friendly services or any services for migrants? When did these services start? What are the activities? - Does your organization have MHV/MHW services? When did these services start? What are the activities? - What is the number of MHVs/MHWs in your organization? What is the ratio of MHVs/MHWs to migrants? |
| **General organizational support** | - What problems and challenges does your organization have about laws and regulations of employment MHVs/MHWs? Please describe. What are the solutions? - Does your organization face any problems with financial support of MHVs/MHWs? Please describe. What are the solutions? - Can you please describe what processes the organization has for MHV/MHW selection, training and supervision? - For training, how does your organization prepare for instructors, content and training methods? - For MHVs/MHWs services, how does your organization collaborate with stakeholders? Please describe. |
| **Specific organizational support to improve access, understand and appraise health information in MHVs/MHWs (Health literacy)** | - What is your opinion about accessing, understanding and appraising health information among MHVs/MHWs under your supervision? - What is your opinion about organizational support to improve access, understanding and appraisal of health information among MHVs/MHWs under your supervision? |
| **Cultural competency in Health personnel** | - Can you please tell me about your experience of treating migrant workers overall, or the last time you treated a migrant worker?   Prompt: specific illnesses, diseases? Treatment or follow-up?   - Does your organization support cultural competency activities? Please specify. - Does your organization provide any guidelines for treating migrant patients, or patients of different ethnicities? If Yes, please describe.   Prompt: Are these MOPH guidelines, Handbooks, Policy documents   - Have you received any training for dealing with migrant patients? If Yes, please describe.   Prompt: Hospital level training? What did the training involve? |
| **Opinions about strengths/challenges and suggestions** | - (Health professionals) Besides language, what are the main cultural misunderstandings between migrant patients and health workers here?   Prompts: patient understanding about diseases, traditional medicine use, treatment adherence (differences by nationality?)   - (Health professionals) How have you tried to overcome cultural misunderstandings, when it comes to treatment?   Prompts: besides MHV – patient education in the community? Pamphlets, physical gestures?   - What is your opinion about MHVs/MHWs services? What are the strengths/challenges and suggestions? - What is your opinion about supporting for migrant-friendly services in term of accessing, understanding and appraising health information among MHVs/MHWs? What are the strengths/challenges and suggestions? |

**Policymakers:**

| **Topics** | **Questions** |
| --- | --- |
| **Background and structure** | - When did the migrant-friendly service policy or any service policies for migrants start? What are the components under this policy? How have you supported this policy? - When did the MHV/MHW service policy start? What are the components under this policy? How have you supported this policy? |
| **General organizational support** | - What support is provided from the central government to follow migrant-friendly service policy, in term of laws and regulations of employment, finance and governance? Please specify. |
| **Specific organizational support to improve access, understand and appraise health information in MHVs/MHWs (Health literacy)** | - What is your opinion about central government support to improve access, understanding and appraisal of health information in MHV/MHW services? |
| **Opinions about strengths/challenges and suggestions** | - What is your opinion about MHVs/MHWs services? What are the strengths/challenges and suggestions? - What is your opinion about support for migrant-friendly services in term of access, understanding and appraisal of health information among MHVs/MHWs? What are the strengths/challenges and suggestions? |
